# Case Report: Clinical awareness about the effect of laser interstitial thermal therapy on pediatric high-grade brain tumors after radiotherapy

**DOI:** 10.3389/fsurg.2024.1462074

**Published:** 2025-01-17

**Authors:** Sandra Fernandes Dias, Markus F. Oertel, Ana Guerreiro Stücklin, Nicolas U. Gerber, Elisa Colombo, Tristan P. C. van Doormaal, Niklaus Krayenbühl

**Affiliations:** ^1^ Division of Pediatric Neurosurgery, University Children’s Hospital Zurich – Eleonoren Foundation, Zurich, Switzerland; ^2^ Department of Neurosurgery and Clinical Neuroscience Center, University Hospital Zurich, University of Zurich, Zurich, Switzerland; ^3^ Department of Oncology and Children’s Research Center, University Children’s Hospital Zurich – Eleonoren Foundation, Zurich, Switzerland

**Keywords:** ependymoma, laser interstitial thermal therapy, neuromere, pediatric neurosurgery, radiotherapy

## Abstract

The use of magnetic resonance-guided laser interstitial thermal therapy (LITT) for the treatment of brain tumors and epileptic lesions has increased in the field of pediatric neurosurgery. However, very little is known about the effect of LITT on pediatric high-grade tumors that have been previously treated with radiotherapy. We report on two cases of children with an unexpected rapid brain tumor progression after LITT. The first case was an 11-year-old boy with a periventricular metastasis of a recurrent anaplastic ependymoma treated with proton-therapy and radiosurgery. The second case was a 6-year-old girl with a Lynch-syndrome and a recurrence of a mesio-temporo-occipital high-grade glioma admitted to gross total resection, proton-therapy, chemotherapy, bevacizumab and immune checkpoint inhibitor. Due to evidence of tumor progression in both cases, a decision was made to perform LITT. Shortly after the laser ablation, we observed a significant tumor growth along the trajectory of the LITT catheters, accompanied by clinical deterioration. The effect of LITT on pediatric ependymoma and high-grade glioma recurrence after radiotherapy is still unclear. The tumor expansion following LITT in these two patients should drive a deeper awareness of the effect of radiation and LITT on the tumor-environment. The breakage of the morphogenetic boundaries of the neuromeres, to which each tumor was initially confined, through the placement of the LITT catheters should be considered while trying to understand the disease spread mechanisms. Based on the experience of our center, we advise a careful implementation of this technique on pediatric high-grade central nervous system tumors, particularly in recurrent tumors that were previously treated with radiotherapy, until the underlying pathophysiologic mechanism has been better understood.

## Introduction

Magnetic resonance-guided laser interstitial therapy (LITT) is a minimally invasive technique suitable especially for the treatment of highly eloquent and deep-seated brain lesions, including metastasis, gliomas and other pathological entities (1). It can also be used when other therapies failed to achieve disease control, or when the patient does not tolerate open resective brain surgery (2). The applicability of LITT in the treatment of epileptogenic lesions such as hypothalamic hamartomas, focal cortical dysplasia, insular epilepsy and periventricular nodular heterotopias has been widely proved, with some patients even reaching Engel I outcomes (3–9). In the pediatric neuro-oncology field, LITT has been increasingly applied for the treatment of low-grade gliomas (LGG), with good rates of tumor decrease and local disease control (10, 11). To date, the number of pediatric patients treated with LITT for high-grade brain tumors is lower when compared to the LGG group, as well as the response to treatment, with the tumor volume remaining unchanged or even showing disease progression (9, 10). So far no explicit mention to the effect of LITT in pediatric high-grade brain tumors after radiotherapy or radiosurgery has been published.

We report on two pediatric patients with recurrent high-grade tumors that shortly after LITT presented with a significant tumor progression along the trajectories of the LITT catheters. Both children were previously treated with proton therapy, and one patient had received additionally radiosurgery. A summary of the possible mechanisms of disease spread observed is presented.

## Case series

### Case 1: metastatic progression of an anaplastic ependymoma

**Figure 1:**
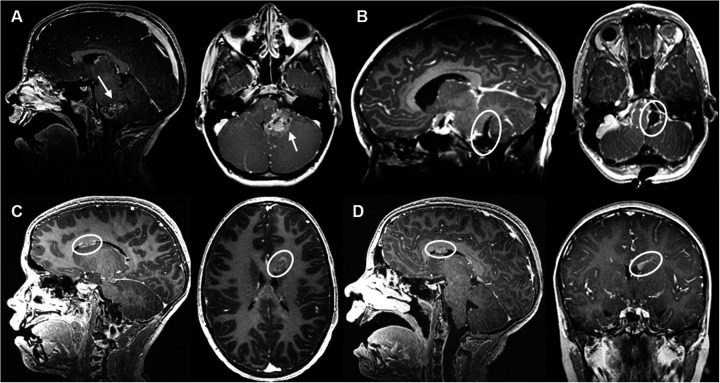
Case 1 sagittal and axial MRI T1 sequences with contrast on follow-up. **(A)** MRI from May 2018 illustrating a space occupying lesion arising from the left foramen of Luschka with contrast-enhancement (arrow). **(B)** Post-surgical resection MRI control revealing gross total resection. **(C)** MRI from December 2020 revealing a new periventricular lesion frontal left, suspected of ependymoma metastasis. **(D)** MRI from April 2021 showing lesion progression after proton-therapy and Cyberknife treatment.

**Figure 2:**
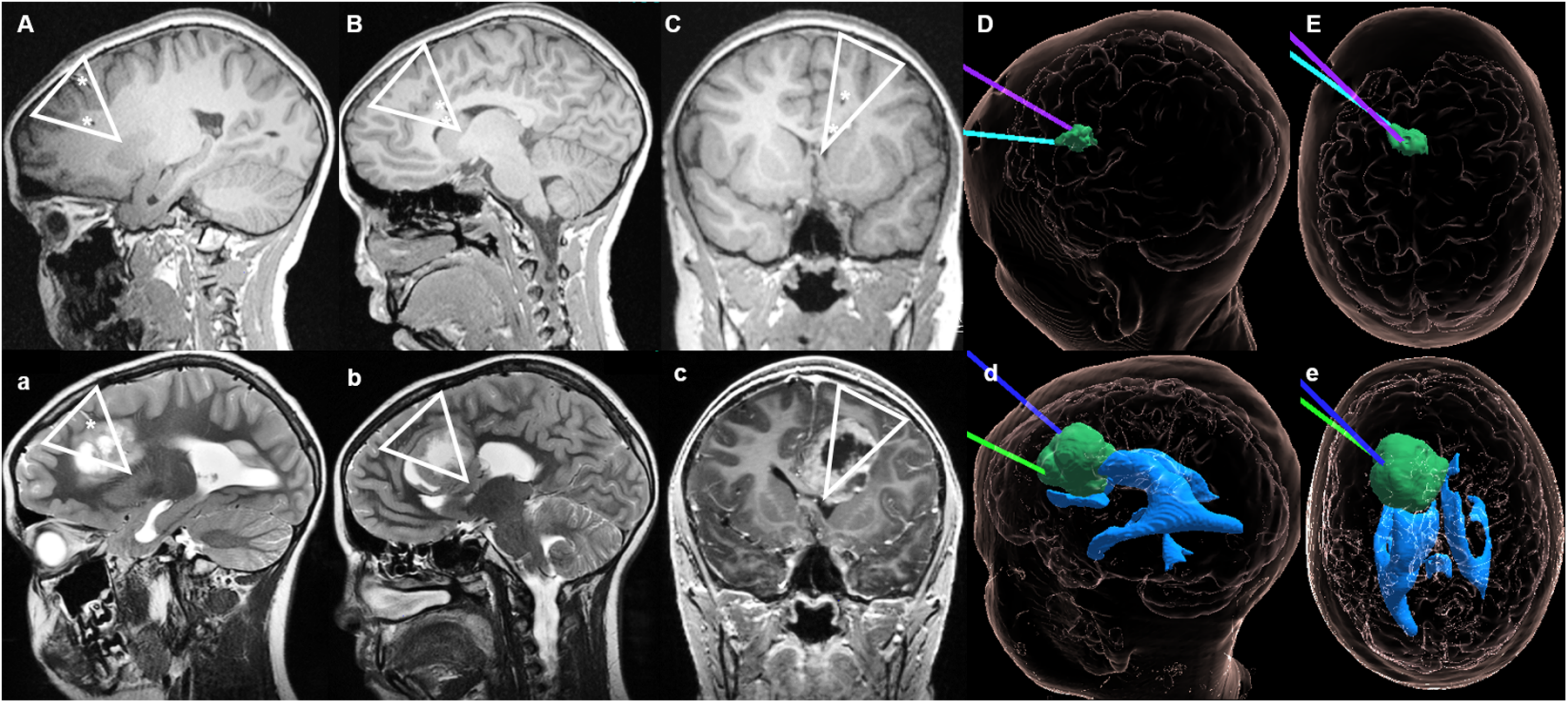
Case 1 sagittal, axial and coronal MRI during LITT and on follow-up. Upper section: **(A)** T1-sequences from May 2021 showing the placement of the catheters for the stereotactic magnetic resonance-guided LITT procedure. **(B)** Sagittal view with both catheters reaching the lesion (final positioning about 1–2 mm deeper). **(C)** Coronal view with the trajectory of the electrodes through the superior and medial frontal gyrus left-sided. **(D)** (sagittal) and **(E)** (axial) hologram (12) views illustrating the relationship between the tumor and position of the two LITT electrodes during treatment. Lower section: **(a)** Tumor expansion observed on MRI from September 2021, in which the direction of growth anatomically correlates with the trajectory of the LITT catheter placed. **(b,d)** Sagittal views showing the tumor expansion along the cingulate gyrus, but with preservation of the cingulate sulcus [which in **(B)** could be seen as not being perforated by the catheter]. **(c)** The triangular shaped tumor growth can also be observed in the coronal view. **(d,e)** Hologram views of the tumor growth along the initial trajectories of the LITT catheters, without invasion of the ventricular system. ▽ on **(A–C)** illustrates the trajectory of the LITT catheters during treatment; ▽ on **(a–c)** shows the pattern of tumor spread after LITT; * represents the location of the LITT catheter on pictures **(A–C)** and the cavity of the LITT's catheter on **(a–c)**.

An 11-year-old boy was operated for non-metastatic posterior fossa (PF) ependymoma (Figure 1A), with a gross total resection being accomplished in May 2018 (Figure 1B). Histopathologic analysis revealed a tumor with fibrillary matrix and perivascular rosettes, GFAP positive and with a Ki67 proliferation index of 80% in some areas, consistent with a PF anaplastic ependymoma, PF-EPN-A, world health organization (WHO) grade 3. As adjuvant treatment, the patient received focal proton therapy in a total dose of 59.4 Gray (Gy). No adjuvant chemotherapy was administered. In December 2019, a new periventricular lesion frontal left (Figure 1C) was found during a magnetic resonance imaging (MRI)-follow-up, suspected to be a metastatic tumor progression. No other metastases were found on craniospinal MRI and lumbar cerebrospinal fluid cytology. The parents declined a surgical approach, and the patient underwent proton irradiation of the craniospinal axis with a total dose 35.2 Gy followed by a stereotactic boost to the metastatic lesion with 5 × 4.5 Gy with Cyberknife linear accelerator. After an initial partial response, a follow-up MRI in April 2021 revealed progression of the same lesion (Figure 1D). The case was discussed in the interdisciplinary neuro-oncologic tumor board. Proton-therapy and Cyberknife had been used without achieving satisfactory disease control. The option to surgically remove the tumor by an interhemispheric approach was discussed. Nevertheless, there was a modest risk of cognitive and concentration problems related to the manipulation of the gyrus cinguli, and parents preferred to avoid open resective surgery. Therefore, a decision in favor of LITT was taken. In May 2021, two catheters were placed along the superior and medial frontal gyrus on the left side (Figures 2A–E). The LITT was performed uneventfully without evidence of complications.

Two months later, a tumor expansion in the left cingulate gyrus and superior frontal gyrus along the trajectory of both previously placed LITT catheters was radiologically observed (Figures 2a–e). Therefore, the patient was submitted to microsurgical resection of the tumor. The patient died in January 2023 due to multifocal tumor progression that was only partially controlled by further palliative Gamma Knife treatments.

### Case 2: recurrent hypermutant high-grade glioma

**Figure 3:**
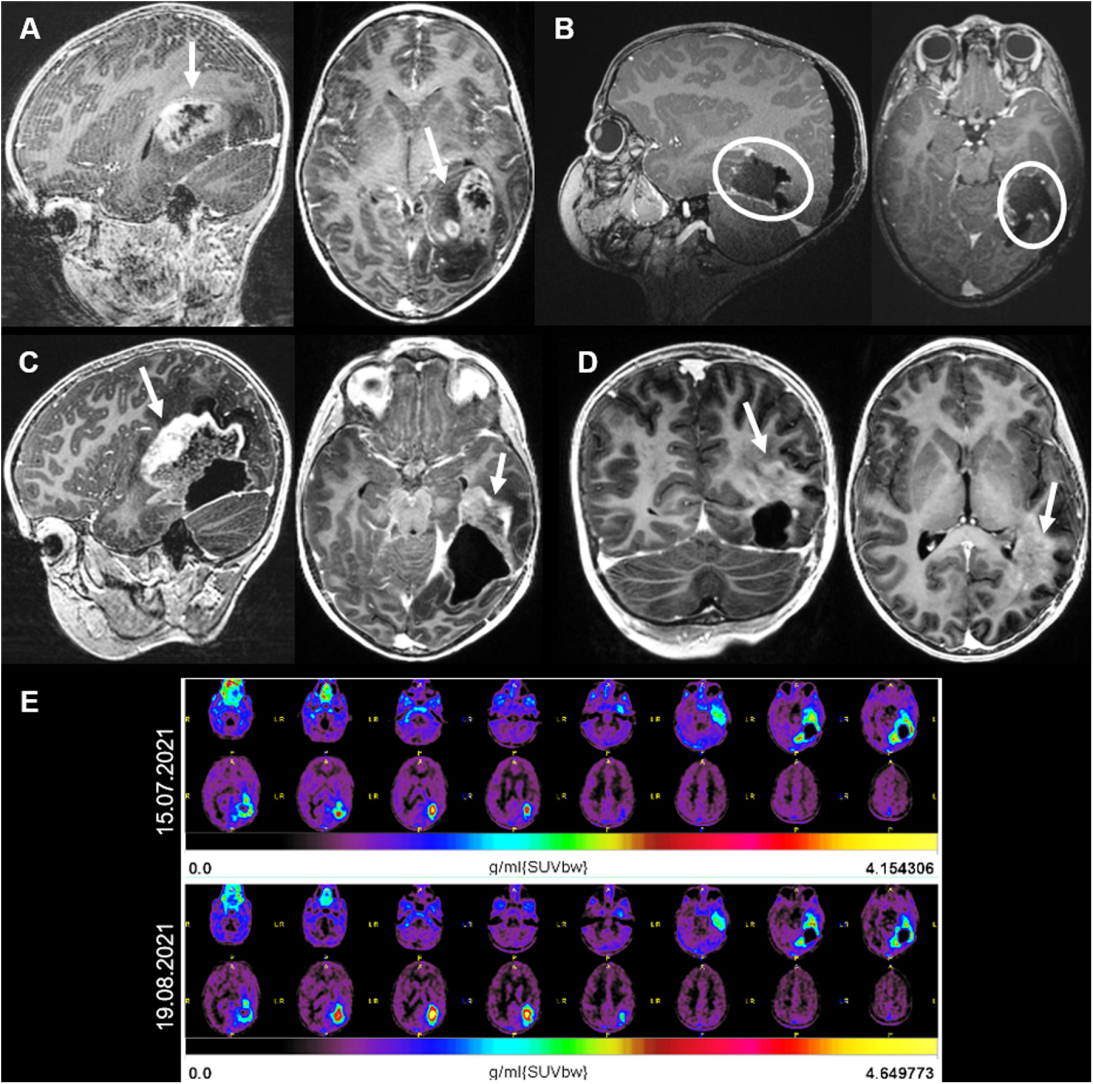
**(A)** Case 2 sagittal, axial and coronal MRI T1 sequences with contrast. **(A)** MRI showing an intra-axial tumor on the left fusiform gyrus with contrast enhancement and central necrosis. **(B)** Postoperative MRI showing a gross total resection. **(C)** Three months after surgery and adjuvant radiotherapy, radio-necrosis reaction (arrows) was superiorly and anteriorly observed. **(D)** One month later, regression of the radio-necrosis but evidence of tumor progression on the left tapetum (arrow). **(E)** ^18^F-fluoroethyltyrosin positron emission tomography-MRIs showing an increase in the FET-activity around the resection cavity, indicating tumor progression.

**Figure 4:**
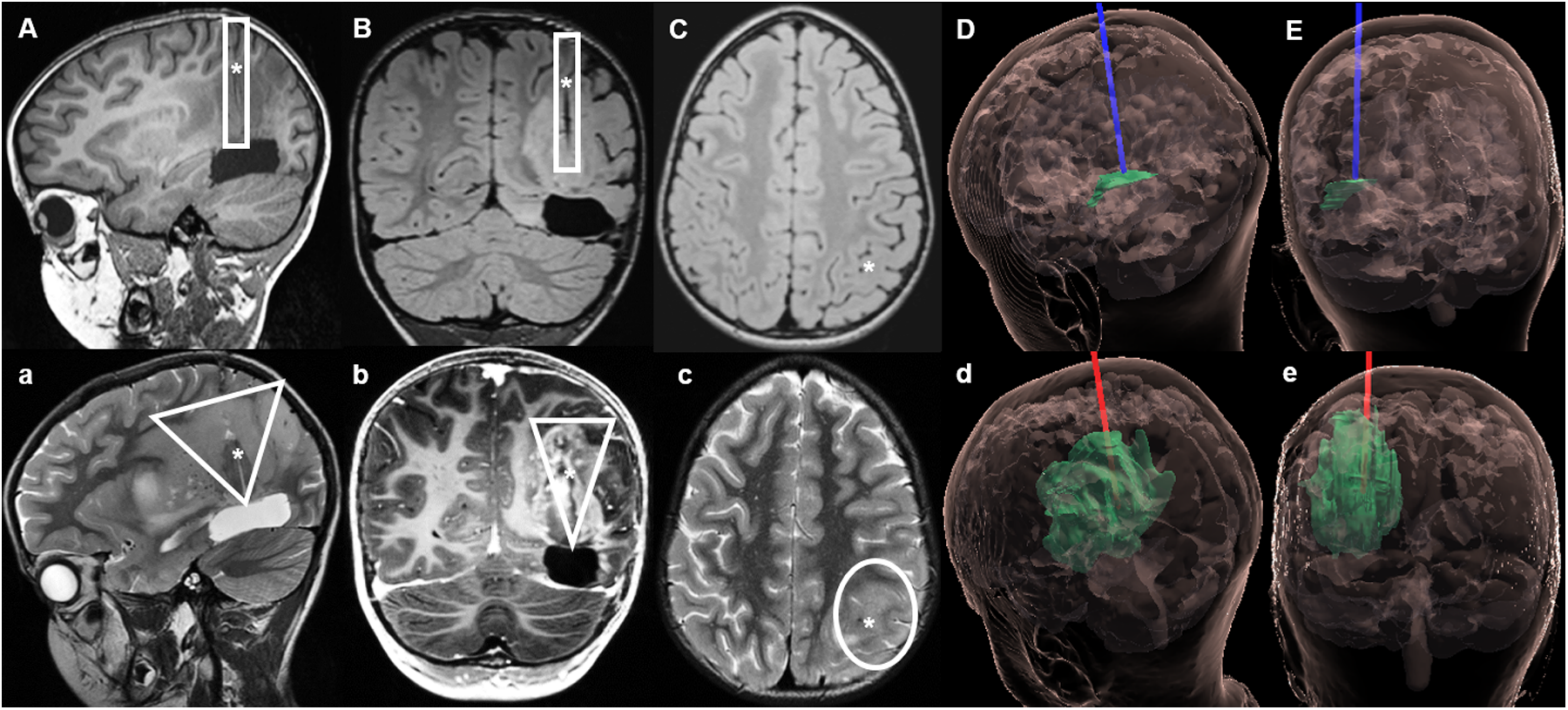
Case 2 sagittal, coronal and axial MRIs during LITT and on follow-up. Upper section: MRI with native T1 sequences during LITT. **(A–C)** The catheter was placed along the left superior parietal lobule, ending on the superior aspect of the resection cavity. **(D)** (sagittal) and **(E)** (coronal) hologram (12) views of the tumor and LITT catheter during treatment. Lower section: Follow-up MRI 2 weeks after LITT, in which a further tumor progression can be observed. **(a,b)** Show a wider tumor spread in a triangular shape along the trajectory of the inserted LITT catheter, with new tumor infiltration on the superficial cortex of superior parietal lobule **(c)**. **(d,e)** Hologram views of the tumor growth along the initial trajectory of the LITT catheter up to the surface. ▯ Illustrates the trajectory of the LITT catheter during treatment; ▽ Shows the pattern of tumor spread after LITT; * represents the location of the LITT catheter on pictures **(A–C)** and the cavity of the LITT catheter on **(a–c)**.

A 6-year-old girl presented with a history of progressive headaches and strabismus. The ophthalmologic examination revealed bilateral papilledema and partial paresis of the left abducens nerve. A brain MRI showed an intra-axial tumor in the left fusiform gyrus with peripheral edema and necrosis (Figure 3A). A treatment with dexamethasone was initiated and 2 days later the patient was operated with near gross total resection being achieved (Figure 3B). The patient was discharged 1 week later with the neurologic examination showing a partial paresis of the left abducens nerve and an incomplete right superior quadrantanopia. Histopathologic analysis revealed a high-grade glioma with loss of MLH1 and PMS2, consistent with a DNA repair deficient-HGG and underlying tumor predisposition. Further genetic testing confirmed the diagnosis of Lynch-syndrome. The child was treated with focal proton therapy and concomitant chemotherapy with lomustine (90 mg/m^2^, oral, every 6 weeks). Due do peri-tumoral brain edema post radiation, bevacizumab was added during radiotherapy (10 mg/kg, every 2 weeks) and initiation of immune checkpoint inhibitors (ICI) deferred. Eight weeks after the end of radiation, the programmed death (PD)-1 inhibitor nivolumab (3 mg/kg, every 2 weeks) was combined to lomustine (90 mg/m^2^) (13, 14). Five months after initial diagnosis, a follow-up MRI showed areas of radionecrosis (Figure 3C) and suspected subcortical tumor progression on the left tapetum (Figure 3D). A positron emission tomography (PET)-MRI (Figure 3E, up), performed to differentiate disease progression from radio-necrosis, showed some tumor activity around the resection cavity, with a progressive recurrence nodule. This was confirmed in a short interval repeated PET-MRI (Figure 3E, down). Due to further tumor progress, the neuro-oncologic tumor-board decided to proceed with LITT. Subsequently, the LITT intervention (Figures 4A–E) was performed without evidence of complications.

An MRI performed 2 weeks later revealed an extensive tumor progression (Figures 4a–e) centered on the ablation channel of the LITT catheter, with newly disease extending to the surface of the superior parietal lobule. Clinically there was a significant deterioration of the child's general condition. A few weeks later, treatment was intensified with a combination of nivolumab (3 mg/kg) with the CTLA4 ICI ipilimumab (1 mg/kg), as well as PARP inhibitor with olaparib (100 mg/m^2^). Unfortunately, the tumor continued to progress, and the child passed away eight months after initial diagnosis.

## Discussion

The use of LITT for the treatment of pediatric brain tumors has been increasing in the last years (9). However the efficacy of LITT in pediatric high-grade gliomas and other malignant CNS tumors that were previously treated with radiation therapy is not known yet. Although LITT has been safely used for the treatment of deep-seated lesions there is always an associated risk of tract seeding. A recent study found a frequency of 5.4% for tumor seeding along the laser fibers after LITT in a mixed pediatric and adult population with brain tumors (15). The three patients in which tract seeding was observed had a median age of 58 years (range 50–60) and were diagnosed with glioblastoma in one case and with brain metastases of melanoma and breast cancer in the other two cases. These patients showed a significantly shorter median time to progression of 1.1 months, compared to the cohort with no tract seeding (4.2 months) (15). The study did not specified how many pediatric patients were treated and which tumor types were involved in this group, but none of them showed tract seeding. Differently to what has been published in the literature, we herein present two cases of recurrent pediatric malignant CNS tumors that showed a significant local tumor progression after LITT with radiological seeding along the trajectory of the LITT catheters. While the mechanism of tumor spread is not entirely understood we aim to elucidate the pathways involved in the disease progression.

### LITT in the treatment of CNS high-grade tumors

In the last decades, there has been an increasing number of studies about the use of LITT for the treatment CNS tumors. A recent series of 313 consecutive adult patients treated with LITT for brain tumors showed that a high-grade lesion was associated with tumor recurrence and decreased overall survival of mean 19.2 months (16). Tract seeding was not mentioned in this study. Traylor et al. studied the effect of LITT in 69 patients with glioblastoma (GBM) in terms of progression-free survival (PFS) and overall survival (OS) (17). Gross total ablation did not significantly improve PFS or OS. Interestingly, adjuvant radiotherapy after LITT slightly prolonged PFS, but it did not affect OS. Meanwhile, the addition of chemotherapy after LITT significantly improved both PFS and OS, pointing the role of LITT in the breakdown of the peritumoral BBB for drug delivery. Similarly, Thomas et al. studied the effect of LITT in eight patients with newly diagnosed GBM and thirteen patients with recurrent GBM (18). In the first group, two patients were submitted to surgical tumor resection within 1 month of LITT and seven underwent radiotherapy and chemotherapy (with temozolamide) after LITT. In the recurrent GBM cohort, twelve patients received radiotherapy and chemotherapy, one patient underwent radiosurgery and two received additional bevacizumab. before LITT. The mean time between diagnosis and LITT was 1 month for the first group and 16 months for the recurrent GBM group. None of the patients with newly diagnosed GBM responded to LITT. In the patient's cohort with recurrent GBM, one third of this patients cohort, which presented with smaller tumor volumes and IDH1 mutation, revealed radiologic tumor volume reduction after laser ablation (18). Whilst the IDH status has not showed so far to be a significant predictor of PFS or OS in GBM patients treated with LITT (17), it remains unclear why a lower response rate to LITT in the patient's cohort with newly diagnosed GBM, was observed. On one hand it could be related to the larger tumor volumes these patients presented, or, on the other hand, to the different therapy protocols applied, as LITT was performed first in the new diagnosed GBM cohort. Whether the shorter time interval between the radiotherapy and the LITT intervention played a role too, as in the recurrent GBM cohort LITT was performed later than 1 year after diagnosis, due to the need for tumor recurrence, remains unclear. Further studies, ideally randomized controlled trials, would be of great interest to further understand the dynamic between the adjuvant therapies and LITT, and determine the optimal time frame to apply this technique. A recent meta-analysis about the use of LITT for the treatment of recurrent GBM confirmed its safety, while revealing a slightly low PFS of 25% at 6 months and 9% at 12 months, and an acceptable OS rate of 92% at 6 months and 42% at 12 months (19). LITT has been applied too in the management of radiation associated necrosis and radiosurgery resistant brain metastases, with good local disease control and weaning of steroids (20, 21). A different approach using LITT as a disrupter of the blood brain barrier (BBB) (22) combined with ICI for the treatment of recurrent IDH-wild-type glioblastoma in adult patients is being explored in a prospective randomized controlled trial (NCT02311582). The first case series of three patients, initially treated with concomitant radiotherapy and temozolomide seem to reveal a positive response (23). While several studies can be found about the effect of LITT on adult high-grade gliomas (HGG), in children, due to their lower occurrence, this disease remains relatively under-investigated (24). Concerning the use of LITT in the pediatric population, Tovar et al. published a series of eleven children, of which two presented with high-grade tumors: one had a thalamic ependymoma which did not receive any previous treatment, and the other child presented with a medulloblastoma recurrence in the brainstem, previously admitted to surgical resection, chemotherapy and stem cell rescue (9). Both patients underwent LITT tumor ablation with no evidence of tumor progression at a follow-up of 32 and 23 months respectively. None of these patients was previously treated with radiotherapy. Slightly contrastingly, a multi-institutional study, which included 76 pediatric patients with low-grade tumors and ten patients with high-grade tumors, revealed that patients with high-grade tumors were more likely to have unchanged or progressing disease after LITT (10).

Even though adult and pediatric HGGs share some tumor drivers (e.g., histone mutations) (24), nowadays they are recognized as distinct entities by the WHO Classification (25), which might partly explains the different responses to treatment observed between adults and children. Furthermore, no studies could be found about the effect of LITT in pediatric high-grade tumors after radiotherapy.

The decision to use LITT in the two cases presented was made at tumor recurrence, with the goal to achieve local disease control using a minimally invasive procedure. Both tumors were treated with proton-therapy prior to LITT, with one patient additionally being submitted to radiosurgery and the other patient being treated with ICI and chemotherapy. There is no published data about the effect of radiotherapy or ICI on the tumor environment when performed before LITT, in the pediatric population. Therefore, we tried to elucidate our findings with the literature available on each aspect that might have contributed to the observed phenomenon.

### Effect of radiotherapy in the tumor microenvironment

Ionizing radiation is known to cause changes in the tumor microenvironment, particularly by damaging the DNA of the surrounding cells, changing the signals transmitted between cells and inducting the production of cytotoxic mediators (26). While the induced apoptosis, arrested tumor cell growth, as well as the enhancement of anti-tumoral immune-response (26, 27) and the regression of non-irradiated metastasis (abscopal effect) (28) are of major relevance for the disease treatment, the destruction of the vascularization in the normal tissue and the induced senescence of non-malignant cells may also have detrimental consequences (26, 29, 30). Recent studies showed that the senescence-associated secretory phenotype (SASP) factors, which consist of a series of inflammatory cytokines, chemokines, growth factors, and matrix remodeling factors that maintain senescent cells in their senescent state of growth arrest, produced by the irradiated surrounding tumor microenvironment may play a significant role in the metastatic spread of disease and local tumor recurrence (29, 31, 32). The dual role of SASP depends on the tumor stage. Whilst SAPS in precancerous epithelial cells can help prevent tumorigenesis, in advanced cancer stages, SAPS in stromal fibroblasts cause tumor progression (32). Fletcher et al. found that eliminating the upregulation of CDKN1A (p21−/−), a relevant promoter of senescence, found in the brain of irradiated mice, could attenuate the growth of glioma cells (31).

The first animal model studies showing microvascular damage and disruption of the BBB after brain radiotherapy report back to the nineties (33, 34). A recent study using both immunocompetent and athymic immunocompromised mice revealed a disruption of the BBB 12 h post whole brain irradiation (WBRT) with a significant increase of the pro-inflammatory cytokines in the brain of the immunocompetent mice (35). Lately, the disruption of the BBB caused by whole brain radiotherapy or radiosurgery has been investigated in patients with brain metastasis using CT and MRI dynamic perfusion imaging techniques (36, 37). By quantifying a transfer constant (K^trans^) during dynamic contrast-enhanced MRI, Teng et al. showed that the vascular permeability in brain metastasis changes after radiotherapy, with significant permeability increase of the blood tumor barrier of metastases that at baseline were low-permeable lesions (37).

We postulate that the occurrence of tumor senescence within and surrounding the tumor nest, as well as the disruption of the BBB after proton therapy and radiosurgery could have contributed to the local progression observed in the two cases herein presented. This was more notorious in the second case (Figure 3C), in which the radio-necrosis seen in the PET-MRI correlated with the extensive tumor spread observed afterwards. The difficulty to differentiate between radio-necrosis and pseudo-progression after radiotherapy in brain tumors persists. While brain perfusion techniques aim to improve the accuracy of diagnosis, they still lack strong validation (38). The correct understanding and interpretation of this phenomena has an added relevance after LITT, as shortly after ablation an increase in the tumor volume is often observed (39). The complexity of the radiological interpretation increases when the patients were previously treated with cranial radiotherapy. Further studies are needed to better understand the changes observed in MRI after LITT in a previously irradiated brain, and to find out which MRI-protocol (or other imaging technique) will provide a better distinction between necrosis, pseudo-progression and true disease progression.

### Tumor phenotype and brain genoarchitecture

In the era of molecular tumor's characterization and classification (40–42), the concept of neural genoarchitecture (43), which refers to the characterization of the neural structures through distinct patterns of gene expression, seems to gain relevance while trying to understand the patterns of tumoral behavior. Previous studies about neurogenesis and CNS segmentation brought us the concept of neuromeres—hierarchically organized ontogenetic units, in which each segment is guided by specific genetic transcripts and carries individual biochemical boundaries (44–46). These initial single layer units further develop during the neurulation process into three dimensional neuro-glial complexes, extending radially from the ventricular to the pial surface of the brain (47).

The concept that each tumor phenotype carries specific molecular characteristics and develops in a defined spatial configuration has become more evident in the last version of the WHO classification of CNS tumors (25). Ependymal tumors are classified according to their molecular subgroup in conjunction with their compartmental occurrence, e.g., supratentorial ependymoma, ZFTA fusion positive, posterior fossa ependymoma group PFA, or spine anaplastic ependymoma (NF2 mutated) (48, 49). The same applies to the group of pediatric diffuse gliomas, in which the midline phenotype is characterized by a H3 K27 modification, while the hemispheric variant shows a H3 G34 mutation (25). This goes in line with the increasing evidence supporting a relationship between the ontogenetic organization of the brain and the phenotype and spatiotemporal distribution of neuroepithelial brain tumors (50).

### Tumor seeding along the LITT tracks

There is a known risk of tumor cells spreading along the trajectory of inserted cannulas or catheters after stereotactic biopsy (51, 52) or ventricular shunt placement (53, 54). In children this phenomenon is observed more frequently with neuroepithelial tumors, in particular, of the pineal region. Furthermore, a recent meta-analysis revealed that in the great majority of the studies (around 78%), a disruption of the peritumoral blood brain barrier (BBB) could be observed after photon therapy (55). Given these premises, the mechanical and thermal opening of the biochemical boundary of the neuro-glial units surrounding the tumor by a biopsy needle or a LITT catheter in a previously disrupted BBB, as a consequence of the proton-therapy or radiosurgery, remains as one plausible explanation for the accelerated tumor progression observed in the two presented cases. Of particular interest is the anatomical distribution of the tumor progression along the LITT catheter trajectories (Figures 2, 4), in regions that were previously radiologically free of tumor. This is notorious in the patient with the anaplastic ependymoma metastasis, as after the tumor had been initially restricted to the left frontal periventricular region for a period of 16 months, confined superiorly by the corpus callosum and cinguli gyrus, shortly after the LITT, a significant disease progression was observed towards the surface, mainly through the left cinguli gyrus and the superior frontal gyrus, along the trajectories in which both catheters were placed (Figures 2a–e). In the second case, although the tumor, previous to LITT, was mostly confined the left tapetum, two weeks after the intervention, a significant tumor progression along the inserted LITT catheter towards the superior parietal lobule was observed (Figures 4a–e), with the tumor then invading this lobule, the parietal operculum and extending to the transverse temporal gyrus.

### Effect of LITT in pediatric high-grade tumors after radiotherapy

This work points to the possibility of rapid tumor expansion after LITT and summarizes a reflection on the feasibly involved mechanisms behind the tumor progression observed in two pediatric cases after LITT has been performed in previously irradiated high-grade CNS tumors. The course of disease observed in these cases raises questions about the mechanism of disease spread and effect of LITT in pediatric high-grade tumors previously submitted to radiotherapy. None of the children were initially treated with chemotherapy. The changes in the tumor microenvironment, with increased permeability of the BBB induced by the previous proton-therapy and radiosurgery, together with the mechanical and thermal disruption of the BBB and neuromeres surrounding the tumor nest may have given a predominant direction of growth and could have possibly contributed to the fast spread of these pediatric high-grade tumors along the catheter's trajectories. However, whether the biochemical boundaries related to the brain ontogenesis initially confine the tumor growth to specific brain regions or the spatiotemporal distribution of the tumor is self-regulated by tumorigenic intrinsic factors remains unknown.

Future research directions should be taken to investigate the changes in the microenvironment of these tumors after radiotherapy and LITT. The performance of molecular analysis and longer follow-up will help us understand the aggressive course of disease observed.

### Limitations

We present two cases of a so far non reported effect of LITT on pediatric high-grade tumors spreading after proton-therapy and radiosurgery. The main limitation of this study is the small number of cases and the short follow-up. This is a consequence of a change in the treatment strategy of pediatric high-grade tumors that we adopted after observing the clinical course of these two cases.

Another aspect is the lack of a separate histology and molecular analysis of the progressing tumor, which could have confirmed the diagnosis and further elucidated the fulminant course of disease. Therefore, the relation between the tumor progression observed and the LITT treatment is based only on the anatomical distribution of the tumor along the trajectory of the LITT catheters seen on the MRI.

## Conclusions

There is a lack of clarity regarding the changes induced by radiotherapy in the tumor microenvironment of pediatric high-grade CNS tumors. The differentiation between radio-necrosis, pseudo-progression or real tumor progression is challenging and despite the improvement in PET and MRI protocols, it is still often difficult to make a clear statement. The effect of LITT on pediatric high-grade tumors following previous treatment, in an altered BBB, needs to be further investigated by molecular analysis of the tumor probes and its microenvironment. Simultaneously, the observation of longer clinical and radiological follow-up would help us to better understand this course of disease and to clarify whether there is an optimal time frame between the end of the radiotherapy and the beginning of LITT treatment. Therefore, a conscientious and critical application of this technique should be considered in pediatric high-grade CNS tumors, when previously treated with proton-therapy and radiosurgery, until the pathophysiologic mechanisms of tumor spread are better understood, and longer follow-up data is available.

## Data Availability

The original contributions presented in the study are included in the article/Supplementary Material, further inquiries can be directed to the corresponding author.
